# Comparison of Efficacy and Safety between Long-Acting Injectable Antipsychotic Monotherapy and Combination of Long-Acting Injectable and Oral Antipsychotics in Patients with Schizophrenia

**DOI:** 10.1155/2021/8403986

**Published:** 2021-11-25

**Authors:** Thanompong Sathienluckana, Pornyupa Tiangpattanawong, Karnpreena Chaiyasukthananoan, Pannapat Jittayanan, Hathaipat Sawetwangsing, Punyawee Puchsaka

**Affiliations:** ^1^Faculty of Pharmacy, Siam University, Bangkok, Thailand; ^2^Department of Somdet Chaopraya Institute of Psychiatry, Thailand

## Abstract

**Background:**

Long-acting injectable (LAI) antipsychotics are used as a monotherapy in patients with schizophrenia. However, the combination of LAI and oral antipsychotics is commonly used in clinical practice, despite there being very limited studies investigating the efficacy and safety of this combination compared with LAI antipsychotic monotherapy.

**Objective:**

To study the efficacy and safety of LAI antipsychotic monotherapy compared with the combination of LAI and oral antipsychotics in patients with schizophrenia.

**Methods:**

This study was a retrospective cohort study, which classified eligible patients into two groups: the LAI antipsychotic monotherapy group and the combination of LAI and oral antipsychotic group. The primary outcome was hospitalization between groups. The duration of the study was 2 years.

**Results:**

In total, 86 patients completed the study and were analysed (LAI antipsychotic monotherapy group: *n* = 25; combination of LAI and oral antipsychotic group: *n* = 61). There was no significant difference in hospitalization between the two groups (*P* = 1.000). For other outcomes, there were also no significant differences in both all-cause discontinuation (*P* = 0.667) and adverse drug reactions (*P* = 0.732) between the two groups.

**Conclusion:**

The efficacy and safety of LAI antipsychotic monotherapy appeared similar to the combination of LAI and oral antipsychotics in patients with schizophrenia. Therefore, the combination of LAI and oral antipsychotics, which is commonly used in clinical practice, may not be necessary.

## 1. Introduction

Schizophrenia is a chronic psychiatric disorder that impairs many aspects of functional outcomes, including social and vocational functioning. Symptom domains of schizophrenia comprise heterogeneous conditions, including positive, negative, cognitive, and affective symptoms [1]. Oral antipsychotics represent the main pharmacological treatment and require long-term use to reduce the risks of relapse and rehospitalization [2]. However, nonadherence to oral antipsychotics is a major and serious problem, which is found in 40–50% of patients with schizophrenia [3]. Nonadherence to oral antipsychotics causes deleterious consequences, including relapse, rehospitalization, longer time to remission, and risk of suicide and substance use. With each relapse, recovery can become slower and less complete, finally leading to the deterioration of the social functioning and living status of patients with schizophrenia [4, 5].

Long-acting injectable (LAI) antipsychotics are an effective treatment strategy in patients with poor adherence to oral antipsychotics, and these medications are used to improve treatment adherence for patients with schizophrenia. LAI antipsychotics are generally recommended as maintenance therapy in chronic schizophrenia [6–8]. Additionally, current research suggests that they may also provide a benefit to patients with early-phase or first-episode schizophrenia [9, 10]. Meta-analysis of mirror-image studies has found that LAI antipsychotics reduce relapse, rehospitalization, and all-cause discontinuation compared to oral antipsychotics [11]. Therefore, most of the guidelines recommend LAI antipsychotics as a monotherapy for relapse prevention in patients with schizophrenia who have a history of nonadherence to oral antipsychotics [2, 8]. However, the concomitant use of LAI and oral antipsychotics is commonly seen in clinical practice in some countries, including Thailand, despite the fact that there have been few studies investigating the efficacy and safety of this combination compared with LAI antipsychotic monotherapy [12–14]. A previous study found that the LAI antipsychotic monotherapy group had a longer time to discontinuation than the combination of LAI and oral antipsychotic group. However, there have not been any studies investigating hospitalization, which was the standard outcome for measuring the efficacy of LAI antipsychotics, for this combination compared with LAI antipsychotic monotherapy. Therefore, this study was aimed at investigating the hospitalization and safety of LAI antipsychotic monotherapy compared with the combination of LAI and oral antipsychotics in patients with schizophrenia.

## 2. Materials and Methods

### 2.1. Study Design and Setting

This was a retrospective cohort study, which collected patients with schizophrenia who received LAI antipsychotics, from outpatient medical records at the Somdet Chaopraya Institute of Psychiatry, Bangkok, Thailand. Eligible patients were classified into two groups: the LAI antipsychotic monotherapy group and the combination of LAI and oral antipsychotic group. Patients were assessed as being in the monotherapy or combination groups after using LAI antipsychotics for 3 months, because most patients were concomitantly using LAI antipsychotics with oral antipsychotics in the first 3 months, which was the overlap period while waiting for a steady state of the LAI antipsychotics. The duration of the study was 2 years.

### 2.2. Participants

Outpatient medical records were reviewed to identify patients who received LAI antipsychotics between January 2012 and December 2016. The inclusion criteria were as follows: (1) age 18–60 years old; (2) had been diagnosed with schizophrenia according to the Diagnostic and Statistical Manual of Mental Disorders, 4^th^ Edition (DSM-IV) or DSM-5; and (3) had received LAI antipsychotics for at least 3 months. Patients were excluded if they had been diagnosed as having treatment-resistant schizophrenia. Treatment-resistant schizophrenia is defined as patients who had no response from two antipsychotics at adequate dose (at least 600 mg of chlorpromazine equivalent) and duration (at least 6 weeks).

### 2.3. Ethics Approval

The study was approved by the Medical Committee of Ethics of the Somdet Chaopraya Institute of Psychiatry.

### 2.4. Outcome Measures

The primary outcome was hospitalization between the LAI antipsychotic monotherapy group and the combination of LAI and oral antipsychotic group. The secondary outcomes included the difference in rates of all-cause discontinuation and adverse drug reactions between the LAI antipsychotic monotherapy group and the combination of LAI and oral antipsychotic group. Reasons for discontinuation were assessed from case notes in outpatient medical records and classified as (1) lack of efficacy, (2) intolerance of adverse drug reactions to medications, (3) clinician decision, or (4) patient decision.

### 2.5. Statistical Analysis

Regarding the sample size, there was no clinical study comparing hospitalization between LAI antipsychotic monotherapy and the combination of LAI and oral antipsychotics in patients with schizophrenia, which made it difficult to set the sample size for determining the differences of hospitalization between groups. Therefore, the study could only extrapolate the sample size from the study by Kane et al., which revealed that treatment with LAI antipsychotics reduced the hospitalization rate when compared with oral antipsychotics. Therefore, a minimum total of 76 patients needed to be recruited for a significance level of *α* = 0.05 and for a power of at least 80% (*β* ≤ 0.20) [15].

Baseline characteristics were compared between groups using Fisher's exact test for nominal data and the Mann-Whitney *U* test for continuous data. Hospitalization, all-cause discontinuation, and adverse drug reactions between the LAI antipsychotic monotherapy group and the combination of LAI and oral antipsychotic group were tested by Fisher's exact test. All analyses were carried out using the Statistical Package for the Social Sciences (SPSS) version 21.

## 3. Results

### 3.1. Patient Characteristics

In total, 86 patients met the inclusion criteria and were included in the analysis (LAI antipsychotic monotherapy group: *n* = 25; combination of LAI and oral antipsychotic group: *n* = 61). Demographic and clinical characteristics of the 86 patients are shown in Table 1. There were no significant differences between treatment groups, including age, sex, marital status, body weight, body mass index, duration of illness, age at onset of illness, psychiatric comorbidities, comedications, type and dose of LAI antipsychotic, and substance use, except for tobacco dependence in patients in the combination of LAI and oral antipsychotic group compared to the LAI antipsychotic monotherapy group (*P* = 0.047). However, there was no correlation between tobacco dependence and hospitalization (*P* = 0.337).

### 3.2. Hospitalization and All-Cause Discontinuation

**Figure 1:**
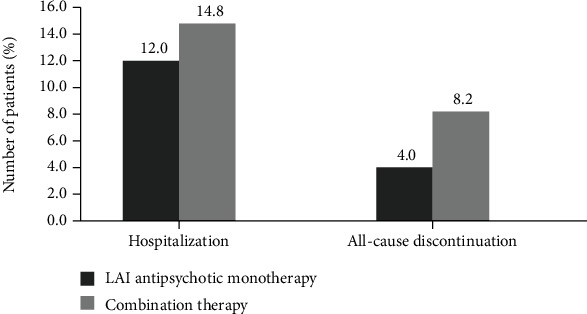
Hospitalization and all-cause discontinuation between the LAI antipsychotic monotherapy and combination therapy groups.

Hospitalization and all-cause discontinuation are shown in Figure 1 and Table 2. There was no significant difference in hospitalization between the LAI antipsychotic monotherapy group and the combination of LAI and oral antipsychotic group (12% vs. 14%; odds ratio [OR] = 0.79; *P* = 1.00). For all-cause discontinuation, there was also no significant difference between the LAI antipsychotic monotherapy group and the combination of LAI and oral antipsychotic group (4% vs. 8.2%; OR = 0.47; *P* = 0.67). For all participants in the study, there were hospitalization and all-cause discontinuation rates of 14% and 7%, respectively, at 2 years.

### 3.3. Adverse Events

Adverse drug reactions between the LAI antipsychotic monotherapy group and the combination of LAI and oral antipsychotic group are shown in Table 3. The most common adverse drug reactions in the study were the extrapyramidal side effect (EPS, 14%) and sedation (9.3%). There were also no significant differences in adverse drug reactions (OR = 1.18; *P* = 0.732) between the LAI antipsychotic monotherapy group and the combination of LAI and oral antipsychotic group.

## 4. Discussion

To the best of our knowledge, this was the first study aimed at comparing the hospitalization between LAI antipsychotic monotherapy and the combination of LAI and oral antipsychotics. This study showed no significant differences in hospitalization and safety between LAI antipsychotic monotherapy and the combination of LAI and oral antipsychotics in patients with schizophrenia. Our study designed this objective because we found that the combination of LAI and oral antipsychotics, which has no strong evidence-based support, is commonly prescribed in clinical practice. A previous study found that about 75% of patients with schizophrenia received the combination of LAI and oral antipsychotics and found no significant difference in the mean clinical global impression-severity (CGI-S) compared with LAI antipsychotic monotherapy [13]. Our study also showed that the combination of LAI and oral antipsychotics, which was prescribed in about 70% (61 of 86) of participants, was common practice and showed no benefit over LAI antipsychotic monotherapy. In contrast to that study, a separate large cohort study of patients with schizophrenia (including treatment-resistant schizophrenia) treated with antipsychotic polypharmacy compared with monotherapy found that antipsychotic polypharmacy was associated with a lower risk of psychiatric rehospitalization. A mentioned possible explanation for these findings relates to the concept of different antipsychotic types and different receptor profiles. Therefore, the combination of LAI and oral antipsychotics with different types of receptor profiles may be considered in some patients with schizophrenia who show an inadequate response to antipsychotic monotherapy. However, our study investigated patients without treatment-resistant schizophrenia. In addition, the combination group of our study originated from the combination of LAI antipsychotics with oral antipsychotics during an overlap period without the gradual discontinuation of oral antipsychotics despite achieving LAI antipsychotic steady state [16].

Our study recruited patients with schizophrenia who had received LAI antipsychotics for at least 3 months, because this period of time was needed for all the LAI antipsychotics included in the study to reach a steady state. The LAI antipsychotics in our study included haloperidol decanoate, fluphenazine decanoate, flupentixol decanoate, risperidone, and paliperidone palmitate, which were all available medications in Thailand at that time. Initial treatment with almost all LAI antipsychotics should overlap with oral antipsychotics (the exception being paliperidone palmitate) to ensure adequate plasma levels of LAI antipsychotics [8, 17]. Haloperidol decanoate had the longest time to steady state (2–3 months) of all the LAI antipsychotics used in the study [7, 17]. Therefore, 3 months after the initiation of LAI antipsychotics is the point at which all oral antipsychotics should have already been discontinued. Thus, our study selected patients who had received LAI antipsychotics for at least 3 months, to compare LAI antipsychotic monotherapy with the combination of LAI and oral antipsychotics. The study excluded treatment-resistant schizophrenia, because treatment with LAI antipsychotics is not recommended as the standard treatment in this patient group [18]. Hospitalization at 2 years was used as a primary outcome, as this is the standard outcome and an adequate duration for studying the treatment of schizophrenia with LAI antipsychotics [19, 20].

In our primary outcome analysis, there was no significant difference in hospitalization between the LAI antipsychotic monotherapy group and the combination of LAI and oral antipsychotic group. This result was consistent with the recommendation of several guidelines for the use of LAI antipsychotics as monotherapy, replacing oral antipsychotics. LAI antipsychotic monotherapy should focus on the use of appropriate doses to achieve a therapeutic effect. The median dose of LAI antipsychotics in both groups was the range of therapeutic dose that had D_2_ receptor occupancy above 60%, which correlates with the therapeutic effect in schizophrenia [20–22]. When compared with previous studies [20], hospitalization at 2 years was about 14% for all patients of our study, which showed the effective treatment with LAI antipsychotics in patients with schizophrenia when used at appropriate doses. For the safety outcome, no significant differences in adverse drug reactions were observed between groups, despite the expectation that the combination group would have more adverse effects compared to the LAI antipsychotic monotherapy group. This result might be related to (1) a small sample size that had no adequate power to detect differences in effects of secondary outcomes or (2) tobacco dependence, which can reduce the plasma level of several antipsychotics due to the CYP1A2-inducing effect being higher in the combination group compared with the monotherapy group [23, 24].

Some limitations of our study should be noted. Firstly, our study was a retrospective cohort study design, which lacks some information such as severity of schizophrenia and baseline number of past hospitalizations. Due to the lack of information about the severity of schizophrenia, the combination group had the possibility of more severe schizophrenia and a higher risk of hospitalization. Moreover, outpatient medical records may not identify whether or not the patient is fully adherent to the oral medication. Secondly, the small number of participants in the study impeded the statistical power to detect significant differences between groups for several outcomes. However, the study reached the sample size estimation for analysis of the primary outcome. Thirdly, most of the LAI antipsychotics in the study were first-generation antipsychotics (FGAs), which may not be generalizable to the results for all LAI antipsychotics. This was related to the impoverished economic status of our patients and to several second-generation antipsychotics (SGAs) not being listed on the National List of Essential Medicines in Thailand. However, the efficacy of LAI antipsychotics is generally similar between FGAs and SGAs.

## 5. Conclusion

This study demonstrates the efficacy and safety of LAI antipsychotic monotherapy, which showed no significant difference when compared with the combination of LAI and oral antipsychotics. Our study was designed to assess hospitalization rates after 2 years, as this has been the standard outcome for measuring the efficacy of LAI antipsychotics in patients with schizophrenia in real life. Our results suggest that the combination of LAI and oral antipsychotics, which is commonly used in clinical practice, may not be necessary. Additional prospective studies with a large sample size are required to further investigate these outcomes.

## Figures and Tables

**Table 1:** Baseline characteristics of the study participants.

Characteristics	LAI antipsychotic monotherapy group (*N* = 25)	Combination of LAI and oral antipsychotics group (*N* = 61)
	Median (IQR)	
Age, years	34.0 (27.0, 47.0)	34.0 (27.0, 41.0)
Body weight, kg	59.2 (50.5, 69.5)	61.2 (56.3, 70.8)
Body mass index, kg/m^2^	23.9 (21.2, 25.0)	24.0 (22.4, 24.3)
Age at onset of illness, years	32.0 (22.8, 41.5)	30.0 (22.2, 35.0)
Duration of illness, years	3.0 (1.0, 4.5)	2.0 (0.5, 7.5)
Dose of LAI antipsychotic, mg (flupenthixol decanoate equivalent)	33.0 (20.0, 63.5)	40.0 (20.0, 50.0)
Dose of oral antipsychotic, mg (chlorpromazine equivalent)	0.0 (0.0, 0.0)	333.0 (200.0, 600.0)
	*N* (%)	
Sex (% male)	10 (40.0)	34 (55.7)
Marital status, % single	16 (64.0)	47 (77.0)
Psychiatric comorbidities, *N* (%)		
Bipolar disorder	0	5 (8.2)
Major depressive disorder	1 (4.0)	2 (3.3)
Catatonia	0	1 (1.6)
Comedications, *N* (%)		
Anti-EPS	20 (80.0)	53 (86.9)
Sedatives/anxiolytics	13 (52.0)	30 (49.2)
Mood stabilizers	2 (8.0)	5 (8.2)
Antidepressants	1 (4.0)	6 (9.8)
Substances, *N* (%)		
Alcohol	6 (24.0)	16 (26.2)
Amphetamine	4 (16.0)	5 (8.2)
Cannabis	0	3 (4.9)
Tobacco^∗^	5 (20.0)	26 (42.6)
Others	1 (4.0)	1 (1.6)
LAI antipsychotics		
Fluphenazine decanoate	2 (8.0)	11 (18.0)
Flupentixol decanoate	14 (56.0)	30 (49.2)
Haloperidol decanoate	6 (24.0)	12 (19.7)
Paliperidone palmitate	3 (12.0)	4 (6.6)
Risperidone	0 (0.0)	4 (6.6)

EPS: extrapyramidal side effect; LAI: long-acting injectable. ^∗^*P* < 0.05.

**Table 2:** Hospitalization and all-cause discontinuation between LAI antipsychotic monotherapy and combination of LAI and oral antipsychotic groups.

Outcomes	LAI antipsychotic monotherapy group (*N* = 25)	Combination of LAI and oral antipsychotic group (*N* = 61)	OR (95% CI)	*P* value
Hospitalization	3 (12.0)	9 (14.8)	0.79 (0.20, 3.19)	1.00
All-cause discontinuation	1 (4.0)	5 (8.2)	0.47 (0.05, 4.21)	0.67
Lack of efficacy	0 (0.0)	1 (1.6)
Clinician decision	1 (4.0)	1 (1.6)
Intolerable of ADR	0 (0.0)	1 (1.6)
Patient decision	0 (0.0)	2 (3.3)

**Table 3:** Adverse drug reactions between the LAI antipsychotic monotherapy and combination of LAI and oral antipsychotic groups.

Adverse drug reactions	LAI antipsychotic monotherapy group (*N* = 25)	Combination of LAI and oral antipsychotic group (*N* = 61)	OR (95% CI)	*P*
Adverse drug reactions, *N* (%)	10 (40.0)	22 (36.1)	1.182 (0.45, 3.07)	0.732
Sedation	1 (4.0)	7 (11.5)
Weight gain	0 (0.0)	1 (1.6)
EPS	4 (16.0)	8 (13.1)
Others	5 (20.0)	6 (9.8)

EPS: extrapyramidal side-effect.

## Data Availability

The data used to support the findings of this study are included within the article.
